# Learning Climate and Job Performance among Health Workers. A Pilot Study

**DOI:** 10.3389/fpsyg.2016.01644

**Published:** 2016-10-25

**Authors:** Michela Cortini, Monica Pivetti, Sara Cervai

**Affiliations:** ^1^Dipartimento di Scienze Psicologiche, della Salute e del Territorio, Università degli Studi “G. d’Annunzio” Chieti - PescaraChieti, Italy; ^2^Dipartimento di Studi Umanistici, Università degli Studi di TriesteTrieste, Italy

**Keywords:** learning climate, job performance, psychological strain, health professions, mediation analysis

## Abstract

This paper will explore if and how psychological strain plays a mediator role between the learning climate and job performance in a group of health workers. Although the relationship between learning climate and job performance has already been explored in the international literature, the role of psychological strain, which may hamper or deepen this relationship, has yet to be investigated. The research hypothesis is that psychological strain mediates the relationship between the climate toward learning (including also the error avoidance climate) and job performance. Data were gathered in a Public hospital in Italy. Participants (*N* = 61) were health professionals (nurses and obstetricians). Considering the relatively small sample size, a mediation analysis with the aid of the SPSS macro PROCESS was performed. The results show that the relationship between the learning climate (specifically its dimension of organizational appreciation toward learning) and job performance is mediated by psychological strain. The future research agenda and practical implications are discussed in the paper.

## Introduction

The relationship between soft constructs (i.e., Perceived Organizational Support, Job Satisfaction, Commitment, Engagement...) and performance has received growing attention from scholars – in particular, work psychology researchers – working to show the link between subjective items (perception, motivation, satisfaction) and measurable outcomes (Riggle et al., 2009).

The dimensions that refer to the psychological side of the organization are – in the common experience of any practitioner – clearly related to personal behaviors and performance, although the scientific literature still struggles to demonstrate it. International contests (e.g., Great Place To Work) are receiving more and more attention from companies, motivated first of all by the need to attract human resources (Employer Branding), and to consider the possibility of a close link between organizational wellbeing and performance.

One open question is about how to demonstrate the relationship between the performance of a whole organization and psychological dimensions such as organizational culture, climate, stress… Managerial studies propose considering economic indicators as proxies for soft constructs: i.e., funds dedicated to training and budget results. Moreover, there is an increasing need to focus on how psychological organizational dimensions can impact on performance.

Organizational performance is usually measured in terms of financial indicators; productivity and performance are often considered good proxies. Furthermore, specific markets and sectors required a specific focus. For instance, in education, where the proposal to measure performance by monitoring the salary of former students, or even by means of financial indicators (ROI), is not well accepted yet, and may not be very appropriate, either.

In the healthcare sector, budget indicators are seen in terms of performance linked to health indicators: healthy people do not require treatment, so they do not cost to the community anything; recovery rates, cure rates, surgery rates, pertinence of prescription are only some examples of how the healthcare sector is used to consider costs and quantitative indicators to measure organizational performance. In this context, incidents and errors may become one of the most appropriate dimensions to analyze the link between organizational dynamics and performance in healthcare. Each incident or error in medicine contains a potential cost in terms of patient health, additional therapies, legal costs, damaged reputation, which can have a direct impact on organizational performance. On the other hand, each incident contains information regarding how to improve procedures to avoid a similar error in the future, so that it constitutes a precious source of information for the continuous improvement process. Generally speaking, errors trigger learning because negative and informative events have the potential to highlight what still needs to be changed, becoming a step in a wide organizational development (Argyris and Schön, 1978). If something changes following an error, it means the organization has learnt from it (Frese and Keith, 2015). Putz et al. (2013, p. 516) proposed a classification in four factors influencing such error- related learning processes at work, underlining that: “While members act and learn as their organization’s agents, the organization itself affects the learning processes of individuals with its routines, processes, structures and its culture.” They consist in supervisor’s behavior, colleagues’ behavior, task structure and procedures, principles and values. Following this rationale, Putz et al. (2013) focus their research on the climate conducive for learning from errors, seen as a multifaceted construct.

Since the error rate can be seen as performance indicator in the healthcare sector (because any error potentially causes a failure), we argue that the way errors trigger an improvement indicates, in a clear way, the organizational learning process, and its effectiveness.

To investigate which dimensions encourage or discourage this process is one of the major challenges for practitioners and researchers.

The link between HR practices and strategies, and in particular training policies, with healthcare organizations’ performance has been reported by Michie and West (2004). Moreover, the relationship between organizational support for training and performance has been well highlighted in the meta-analytic review conducted by Arthur et al. (2003).

More recently, Nikolova et al. (2014) have reviewed the organizational learning literature, and have underlined three different dimensions that occur in different studies and different contexts; the facilitation dimension (all the forms of support for learning that organizations guarantee to their employees); the dimension of rewarding and appreciation for learning, such as incentives; the dimension of error management. These dimensions have been included in the Learning Climate Scale (LCS).

To contribute to this field of research, the present study focuses on the relationship between learning climate and performance, seeking to understand the influence played by an individual dimension, particularly challenging nowadays, that is psychological strain.

In other studies (Cervai et al., 2013; Cervai and Polo, 2015) we have stressed the fact that learning is crucial for the health context, stressing the need to evaluate its outcome. In line with this attention toward learning outcome evaluation, we propose to investigate the role of the internal evaluation of learning. In particular, the research aims to explore the role played by the organizational learning climate, which can become a proxy to evaluate workplace learning.

In particular, following the work of Motowidlo et al. (1986), our attention focuses on the mediator role of psychological strain between learning climate and performance, putting forward the following hypothesis:

H1: Perceived organizational support for training and learning positively affects job performance.
H2: Appreciation from the organization for training and learning activities positively affects job performance.
H3: Error avoidance negatively affects job performance.
H4: Psychological strain mediates the relationship between support for training and learning and job performance.
H5: Psychological strain mediates the relationship between appreciation for training and learning and job performance.
H6: Psychological strain mediates the relationship between error management and job performance.

## Materials and Methods

### Sample

Data were gathered in a Public hospital in Italy. Participants (*N* = 61) were full-time health professionals (nurses and obstetricians); most of them (51 participants) were female, their average age was 47.9 years (SD. 9,6), with a range between 31 and 68 years of age.

### Measures and Data Collection

A cross-sectional survey was adopted with a self-administered and anonymous questionnaire, with a first part on socio-demographic information (age, sex, contract type) and a core part related to the variables being analyzed whose description follows.

#### Learning Climate

The original scales developed by Nikolova et al. (2014) were used to assess the following dimensions of the learning climate: organizational support for training (ex. “My organization provides sufficient resources to develop my competences”), appreciation for training (e.g., “In my organization, employees who make effort to learn new things, earn appreciation and respect”) and error avoidance (e.g., “In my organization, one is afraid to admit mistakes”). These measures showed high Cronbach alpha reliability levels (indexes vary from 0.81 to 0.91). All the scales consist of three items where respondents express an opinion by means of a seven-point Likert scale, representing an increasing level of agreement (from 1, “strongly disagree” to 7, “strongly agree”).

#### Psychological Strain

Job strain was measured with a single item of the General Health Questionnaire (GHQ) focused on feelings of stress. The GHQ has a long tradition within nursing studies (e.g., Hunter and Houghton, 1993; Fagin et al., 1995; Jones and Johnston, 1997; Tully, 2004; Gomes et al., 2013). Furthermore, in the past, the GHQ was used as a simple tool for detecting the prevalence of psychological stress within general medical practice (Werneke et al., 2000). Several versions of the GHQ exist: 60-, 30-, 28-, and 12-item scales. GHQ-12 is perhaps the most popular version of the questionnaire, very often used in large social surveys (Banks and Jackson, 1982; Winefield et al., 1989) or applied contexts (Tully, 2004), owing to its brevity.

The choice of the single item was driven by a cost and time rationale, especially in view of the fact that we had collected data inside the working context, in the time between shifts. The choice to replace longer measurement scales with a single measure is not a novelty in the international literature and it seems to be particularly efficient for the working environment, showing a satisfactory content validity (see, Elo et al., 2003). We have preferred the single item taken from the GHQ (“Have you experienced stress during the past 2 weeks?”, measured by a four-point scale, ranging from 1, “better than usual,” to 4, “Much less than usual”), instead of the one derived from the Occupational Stress Questionnaire (Elo and Leppänen, 1999), because the latter includes an introduction (“Stress means a situation in which a person feels tense, restless, nervous or anxious or is unable to sleep at night because his/her mind is troubled all the time. Do you feel this kind of stress these days?”) that might, in our opinion, risk confusing the respondents. We believe that nowadays people are so used to talking about stress that they do not need any kind of detailed explanation to understand whether they are experiencing stress or not.

#### Job Performance

Finally, in line with the most recent research in work and organizational psychology (e.g., Demerouti et al., 2005), we have decided to use a self-rated job performance measure, adopting two items from Chirumbolo and Areni (2005). The first item was “I have achieved all my job goals in the last 6 months” to which participants had to declare their agreement/disagreement (1 = strongly disagree; 9 = strongly agree). The second item was “In the last 6 months, your job performance was:” to which participants had to answer using a five-point scale ranging from 1 = particularly low, to 9 = particularly high.

We have decided to use self-reported measures of the main study dimensions, since we focused on *perceived* job characteristics (job satisfaction, as well as learning climate), as many other researchers have done (Chan, 2009; Judge et al., 2000), “making self-reports the theoretically most relevant measure method” (Conway and Lance, 2010, p. 329).

## Results

None of the demographic variables related to any of the study variables so they were excluded from further analysis. **Table 1** presents the preliminary analysis of the dataset: the means, standard deviations and inter-correlations of the main study variables.

**Table 1:** Descriptive statistics and correlations.

	NI	*M*	*SD*	Alpha	2	3	4	5
(1) Psychological strain	1	2.26	1.04	–	-0.429^∗∗^	-0.266^∗^	0.110	-0.375^∗∗^
(2) Organizational support for training	3	2.75	1.15	0.91		0.569^∗∗^	0.120	0.550^∗∗^
(3) Appreciation for training	3	2.17	0.94	0.83			-0.043	0.600^∗∗^
(4) Error avoidance	3	3.51	0.86	0.86				0.077
(5) Performance	2	4.80	1.85	0.76				

*n = 61; NI, number of items; ^∗^*p* < 0.05; ^∗∗^*p* < 0.01.*

Firstly, scaled variables achieved good reliability, with Cronbach’s alpha coefficients between 0.76 and 0.91.

### Test Hypotheses

In order to test H1, H2, and H3 we performed a multiple linear regression, to predict job performance based on the three different dimensions of the Learning Climate: organizational support for training, appreciation for training and error avoidance. Preliminary analysis was performed to ensure there was no violation of the assumption of normality, linearity and multicollinearity.

A significant regression equation was found: *F*(1,59) = 14,22; *p* < 0.001, with an *R*^2^ = 0.42.

Only organizational support and training appreciation were significant predictors (**Table 2**) of job satisfaction for apprentices.

**Table 2:** Summary of simple regression analysis for learning climate dimensions predicting job satisfaction (*N* = 86); ^∗^*p* < 0.05; ^∗∗∗^*p* < 0.001.

	*B*	*SE B*	β
Organizational support for training	0.507	0.212	0.295^∗^
Appreciation for training	0.789	0.223	0.435^∗∗∗^
Error Avoidance	0.119	0.203	0.060
*F* = 14.22^∗∗∗^			
*R*^2^ = 0.42			

We decided to go further and test, using three independent mediation models, the power of psychological strain to mediate the relationship between all the learning climate dimensions and job performance.

### Mediation Analysis

There are two different methods for performing mediation analyses: Multiple Regression and Structural Equations Models (SEM). Although some researchers (Baron and Kenny, 1986; Hoyle and Smith, 1994; Kenny et al., 1998) suggest adopting SEM because it allows for a better control of measurement error and gives good information about the fit of the model, we follow the guidelines of those (Holmbeck, 1997; Frazier et al., 2004) who suggest using Multiple Regression in case of a small sample size, as in the present study.

We tested the hypotheses following the guidelines described by Preacher and Hayes (2004), who developed an SPSS macro, called PROCESS, that triangulates the normal theory approach (i.e., the Sobel test), a bootstrap approach, and Baron and Kenny’s (1986) approach to measure the indirect effects of the predictor on the dependent variable. In particular, the use of bootstrapped confidence intervals was necessary in order to avoid problems related to our limited sample size (MacKinnon et al., 2004; Preacher et al., 2007) and to take into consideration also the potential mediation for the dimension of error avoidance, which definitely seems interesting for this research, and it is not among the significant predictors of job performance in the present regression analysis.

We performed three different mediation analyses for each of the dimensions of the Learning Climate, with the aid of the macro PROCESS, using model n. 4.

**FIGURE 1:**
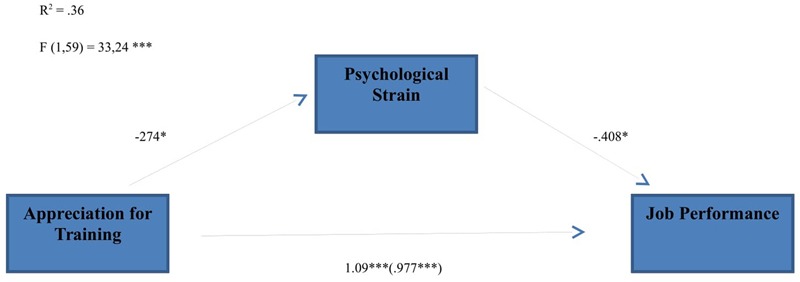
**Indirect effect of organizational appreciation for training on job performance through psychological strain; ^∗^*p* < 0.05; ^∗∗^*p* < 0.01; ^∗∗∗^*p* < 0.001**.

First, it was found that psychological strain significantly mediated the relation between appreciation for training and job satisfaction (**Figure 1**) (indirect effect = 0.112, *SE* = 0.108, 95% CI [0.0109,0.2840]); since zero is not in the 95% confidence interval we can conclude that the indirect effect is significantly different from zero at *p* < 0.05, and that, as predicted, change in psychological strain mediates the relationship between training support and job satisfaction.

The mediator could account for an almost 10% of the effect; *P*M = 0.11; *K*^2^ = 0.07, CI [0.0110,0.1875]).

Even if the role of mediator is not crucial as it would be in a totally mediated output, the interactive force between stress and valorization of learning and training is worth underlining, which suggests to HR managers the importance of taking into account nurses’ training needs.

It was also found that psychological strain did not significantly mediate the relationship between organizational support for training and job performance or the relationship between error avoidance and job performance.

## Discussion

First of all, the regression analysis clearly underlined the dimension of learning as being crucial in terms of performance. Learning, in this sense, has to be understood not only as something that happens at an individual level but rather as something involving employer responsibilities. In particular, the Climate for Learning underlines that, on the one hand, giving support for training and learning and, on the other hand, valuing the learning activities and efforts made by every single worker, is something vital (we suppose in both instrumental and motivational terms) for performance development.

In addition, the significant mediation model provides support for the prediction that nurses experience differences in job performance due to their different levels of psychological stress and the valorization they perceive they receive from their organization. Consequently, these results suggest a series of guidelines for HR managers, who should develop specific training plans and seek to appreciate every single learning outcome, with relentless attention paid to the wellbeing and stress of their employees: in line with other scholars (Montani et al., 2015; Giorgi et al., 2015) we claim for a supervisors’ empowering management practices

In terms of additional practical implications, notwithstanding the importance of both learning and training, on the one hand, and stress and well-being, on the other one, we trust the potential of learning and training activities focused on stress management. Stress management, generally being paid for by the employees is not sufficiently promoted within organizations. In terms of primary prevention, we wonder about the role of specific devices and tools (think to the organizational gym) to prevent psychological strain; something that could play a role on performance.

Finally, our results may corroborate the proposals for HR managers we have put forward in other studies (Cortini, 2016), stressing the role of organizational support in order to develop a sense of self as worker; in such a sense, employers ought to provide participatory practices to discuss work-related issues together along with proper error management that allow errors to become a form of learning.

## Limitations and Future Research

The results of the present study should be seen in the light of some limitations. First of all, our study has a cross-sectional design, which limits our capability to extrapolate the causal relationship between the variables under investigation. Especially in view of the recent criticism cross-sectional mediation analysis has received (e.g., Maxwell and Cole, 2007; Mitchell and Maxwell, 2013), we plan to collect longitudinal data to be analyzed by a Continuous Time Mediation, as suggested by Deboeck and Preacher (2016) in order to respond to the limit of mediation models in terms of time-lag analysis.

Furthermore, we have used a small sample size, which should definitely be enlarged. Specifically, our data were collected in a single Italian hospital; it would be necessary to collect additional data, also qualitative in nature, so as to deepen both the relationships we did find significant as well as those we did not find to be significant (but still showing a trend in the hypothesized direction). In addition, with a larger sample it may be easier to check for differences in formal and informal learning, as done by other scholars (Kyndt et al., 2009; Manuti et al., 2015).

We have decided to focus on self-rated performance; it would be interesting in the upcoming future to detail performance and to distinguish between adaptive performance and task performance, in line with recent literature trends (Shoss et al., 2012; Jundt et al., 2015).

Finally, taking into consideration our results, we suggest a venue for future studies that will allow for a greater understanding of the processes of workplace learning in the health context. In particular, these recommendations include further investigation of the different roles the managers and the organizational climate may play, as well as assessing the potential outcomes at both organizational and patient levels.

## Author Contributions

SC and MC designed the pilot study. SC and MC reviewed literature. MP collected data. MC analysed data. Together, MC, SC, and MP wrote discussion, limitations and future research agenda

## Conflict of Interest Statement

The authors declare that the research was conducted in the absence of any commercial or financial relationships that could be construed as a potential conflict of interest.

The reviewer JP and the handling Editor declared their shared affiliation, and the handling Editor states that the process nevertheless met the standards of a fair and objective review.

## References

[B1] Argyris C. Schön D. A. (1978). *Organizational Learning: A Theory of Action Perspective*. Reading, MA: Addison-Wesley.

[B2] Arthur W. Jr. Bennett W. Jr. Edens P. S. Bell S. T. (2003). Effectiveness of training in organizations: a meta-analysis of design and evaluation features. *J. Appl. Psychol.* 88 234–245. 10.1037/0021-9010.88.2.234 12731707

[B3] Banks M. H. Jackson P. R. (1982). Unemployment and risk of minor psychiatric disorder in young people: cross-sectional and longitudinal evidence. *Psychol. Med.* 12 789–798. 10.1017/S0033291700049096 6984196

[B4] Baron R. M. Kenny D. A. (1986). The moderator-mediator variable distinction in social psychological research: conceptual, strategic, and statistical considerations. *J. Pers. Soc. Psychol.* 51 1173–1182. 10.1037/0022-3514.51.6.1173 3806354

[B5] Cervai S. Cian L. Berlanga A. Borelli M. Kekäle T. (2013). Assessing the quality of the learning outcome in vocational education: the Expero model. *J. Workplace Learn.* 25 198–210. 10.1108/13665621311306565

[B6] Cervai S. Polo F. (2015). Evaluating the quality of the learning outcome in healthcare sector: the Expero4care model. *J. Workplace Learn.* 27 611–626. 10.1108/JWL-09-2015-0063

[B7] Chan D. (2009). “So why ask me? Are self-report data really that bad,” in *Statistical & Methodological Myths and Urban Legends: Doctrine, verity and fable in the organizational and social sciences* eds Lance C. E. Vandenberg R. J. (Hillsdale, NJ: Erlbaum) 309–336.

[B8] Chirumbolo A. Areni A. (2005). The influence of job insecurity on job performance and absenteeism: the moderating effect of work attitudes. *SA J. Ind. Psychol.* 31 65–71. 10.4102/sajip.v31i4.213

[B9] Conway J. M. Lance C. E. (2010). What reviewers should expect from authors regarding common method bias in organizational research. *J. Bus. Psychol.* 25 325–334. 10.1007/s10869-010-9181-6

[B10] Cortini M. (2016). Workplace identity as a mediator in the relationship between learning climate and job satisfaction during apprenticeship: suggestions for HR practitioners. *J. Workplace Learn.* 28 54–65. 10.1108/JWL-12-2015-0093

[B11] Deboeck P. R. Preacher K. J. (2016). No need to be discrete: A method for continuous time mediation analysis. *Struct. Equ. Modeling* 23 61–75. 10.1080/10705511.2014.973960

[B12] Demerouti E. Verbeke W. J. Bakker A. B. (2005). Exploring the relationship between a multidimensional and multifaceted burnout concept and self-rated performance. *J. Manag.* 31 186–209. 10.1177/0149206304271602

[B13] Elo A. L. Leppänen A. (1999). Efforts of health promotion teams to improve the psychosocial work environment. *J. Occup. Health Psychol.* 4 87–94. 10.1037/1076-8998.4.2.87 10212862

[B14] Elo A. L. Leppänen A. Jahkola A. (2003). Validity of a single-item measure of stress symptoms. *Scand. J. Work Environ. Health* 29 444–451. 10.5271/sjweh.752 14712852

[B15] Fagin L. Brown D. Bartlett H. Leary J. Carson J. (1995). The Claybury community psychiatric nurse stress study: is it more stressful to work in hospital or the community? *J. Adv. Nurs*. 22 347–358. 10.1046/j.1365-2648.1995.22020347.x 7593957

[B16] Frazier P. A. Tix A. P. Barron K. E. (2004). Testing moderator and mediator effects in counseling psychology research. *J. Couns. Psychol.* 51 115–134. 10.1037/0022-0167.51.1.115

[B17] Frese M. Keith N. (2015). Action errors, error management, and learning in organizations. *Annu. Rev. Psychol.* 66 661–687. 10.1146/annurev-psych-010814-015205 25251490

[B18] Giorgi G. Mancuso S. Perez F. J. F. Montani F. Courcy F. Arcangeli G. (2015). Does leaders’ health (and work-related experiences) affect their evaluation of followers’ stress? *Saf. Health Work* 6 249–255. 10.1016/j.shaw.2015.07.005 26929835 PMC4674506

[B19] Gomes S. F. S. Santos M. M. M. Carolino E. T. D. M. (2013). Psycho-social risks at work: Stress and coping strategies in oncology nurses. *Lat. Am. J. Nurs.* 21 1282–1289. 10.1590/0104-1169.2742.2365 24271316

[B20] Holmbeck G. N. (1997). Toward terminological, conceptual, and statistical clarity in the study of mediators and moderators: examples from the child-clinical and pediatric psychology literatures. *J. Consult. Clin. Psychol.* 65 599–610. 10.1037/0022-006X.65.4.599 9256561

[B21] Hoyle R. H. Smith G. T. (1994). Formulating clinical research hypotheses as structural equation models: a conceptual overview. *J.Consult. Clin. Psychol.* 62 429–440. 10.1037/0022-006X.62.3.429 8063970

[B22] Hunter P. Houghton D. M. (1993). Nurse teacher stress in northern Ireland. *J. Adv. Nurs.* 18 1315–1323. 10.1046/j.1365-2648.1993.18081315.x 8376671

[B23] Jones M. C. Johnston D. W. (1997). Distress, stress and coping in first-year student nurses. *J. Adv. Nurs.* 26 475–482. 10.1046/j.1365-2648.1997.t01-5-00999.x 9378866

[B24] Judge T. A. Bono J. E. Locke E. A. (2000). Personality and job satisfaction: the mediating role of job characteristics. *J. Appl. Psychol.* 85 237–249. 10.1037/0021-9010.85.2.237 10783540

[B25] Jundt D. K. Shoss M. K. Huang J. L. (2015). Individual adaptive performance in organizations: a review. *J. Organ. Behav.* 36 S53–S71. 10.1002/job.1955

[B26] Kenny D. A. Kashy D. A. Bolger N. (1998). “Data analysis in social psychology,” in *Handbook of Social Psychology* 4th Edn eds Gilbert D. Fiske S. T. Lindzey G. (New York, NY: McGraw-Hill) 233–265.

[B27] Kyndt E. Dochy F. Nijs H. (2009). Learning conditions for non-formal and informal workplace learning. *J. Workplace Learn.* 21 369–383. 10.1108/13665620910966785

[B28] MacKinnon D. P. Lockwood C. M. Williams J. (2004). Confidence limits for the indirect effect: Distribution of the product and resampling methods. *Multivariate Behav. Res.* 39 99–128. 10.1207/s15327906mbr3901_4 20157642 PMC2821115

[B29] Manuti A. Pastore S. Scardigno A. F. Giancaspro M. L. Morciano D. (2015). Formal and informal learning in the workplace: a research review. *Int. J. Train. Dev.* 19 1–17. 10.1111/ijtd.12044

[B30] Maxwell S. E. Cole D. A. (2007). Bias in cross-sectional analyses of longitudinal mediation. *Psychol. Methods* 12 23–44. 10.1037/1082-989X.12.1.23 17402810

[B31] Michie S. Atkins L. West R. (2014). *The Behaviour Change Wheel: A Guide to Designing Interventions*. London: Silverback Publishing.

[B32] Mitchell M. A. Maxwell S. E. (2013). A comparison of the cross-sectional and sequential designs when assessing longitudinal mediation. *Multivariate Behav. Res.* 48 301–339. 10.1080/00273171.2013.784696 26741846

[B33] Montani F. Courcy F. Giorgi G. Boilard A. (2015). Enhancing nurses’ empowerment: the role of supervisors’ empowering management practice. *J. Adv. Nurs.* 71 2129–2141. 10.1111/jan.12665 25869300

[B34] Motowidlo S. J. Packard J. S. Manning M. R. (1986). Occupational stress: its causes and consequences for job performance. *J. Appl. Psychol.* 71 618–629. 10.1037/0021-9010.71.4.618 3804934

[B35] Nikolova I. Van Ruysseveldt J. De Witte H. Van Dam K. (2014). Learning climate scale: construction, reliability and initial validity evidence. *J. Vocat. Behav.* 85 258–265. 10.1016/j.jvb.2014.07.007

[B36] Preacher K. J. Hayes A. F. (2004). SPSS and SAS procedures for estimating indirect effects in simple mediation models. *Behav. Res. Methods Instrum. Comput.* 36 717–731. 10.3758/BF03206553 15641418

[B37] Preacher K. J. Rucker D. D. Hayes A. F. (2007). Addressing Moderated Mediation Hypotheses: theory, methods, and prescriptions. *Multivariate Behav. Res.* 42 185–227. 10.1080/00273170701341316 26821081

[B38] Putz D. Schilling J. Kluge A. Stangenberg C. (2013). Measuring organizational learning from errors: development and validation of an integrated model and questionnaire. *Manag. Learn.* 44 511–536. 10.1177/1350507612444391

[B39] Riggle R. J. Edmondson D. R. Hansen J. D. (2009). A meta-analysis of the relationship between perceived organizational support and job outcomes: 20 years of research. *J. Bus. Res.* 62 1027–1030. 10.1016/j.jbusres.2008.05.003

[B40] Shoss M. K. Witt L. A. Vera D. (2012). When does adaptive performance lead to higher task performance? *J. Organ. Behav.* 33 910–924. 10.1002/job.780

[B41] Tully A. (2004). Stress, sources of stress and ways of coping among psychiatric nursing students. *J. Psychiatr. Ment. Health Nurs.* 11 43–47. 10.1111/j.1365-2850.2004.00682.x 14723638

[B42] Werneke U. Goldberg D. P. Yalcin I. Üstün B. T. (2000). The stability of the factor structure of the General Health Questionnaire. *Psychol. Med.* 30 823–829. 10.1017/S0033291799002287 11037090

[B43] Winefield H. R. Goldney R. D. Winefield A. H. Tiggemann M. (1989). The General Health Questionnaire: reliability and validity for Australian youth. *Aust. N. Z. J. Psychiatry* 23 53–58. 10.3109/00048678909062592 2930415

